# Effects of a 12-Weeks Yoga Intervention on Motor and Cognitive Abilities of Preschool Children

**DOI:** 10.3389/fped.2021.799226

**Published:** 2021-11-26

**Authors:** Aleksandra Aleksić Veljković, Borko Katanić, Bojan Masanovic

**Affiliations:** ^1^Faculty of Sport and Physical Education, University of Niš, Niš, Serbia; ^2^Faculty for Sport and Physical Education, University of Montenegro, Nikšić, Montenegro; ^3^Montenegrosport, Podgorica, Montenegro

**Keywords:** motor skills, cognitive function, physical development, physical activity, coordination

## Abstract

Since early childhood is regarded as an important period of motor and cognitive development, understanding the effects of physical activity on motor abilites and cognitive development in preschool children has major public health implications. This study investigates the effects of a 12 weeks' yoga intervention program on motor and cognitive abilities in preschool children. Preschool children (*n* = 45; age 5–6 years) attending regular preschool programs were non-randomly assigned to yoga intervention (*n* = 23; 30 min sessions three times per week) or a control group (*n* = 22; no additional organized physical activity program). Exercise training for the intervention group included yoga program. Motor abilities (BOT-2 subtests: fine motor integration, manual dexterity, balance and bilateral coordination), and cognitive abilities (School Maturity Test subtests: visual memory, stacking cubes and codes) were assessed before and after the intervention period in both groups. Data were analyzed using repeated-measures ANOVA. Participants in the intervention group improved fine motor integration (*p* = 0.022), fine motor skills in general (0.029), bilateral coordination (0.000), balance (0.000), and body coordination (0.000). Preschool children's participation in the preschool yoga intervention significantly improved their motor abilities, but not their cognitive abilities.

## Introduction

Since early childhood is regarded as an important period of motor and cognitive development, understanding the effects of physical activity on motor abilities and skills, and cognitive development in preschool children has major public health implications (1). Regular participation in physical activities prevents obesity in children and provides numerous health benefits, both physical psychological (2). Along with prevention and reduction of obesity in children, physical activity is also connected with health and good condition of vascular system, muscle strength and endurance, reduction of depression and anxiety, as well as with the academic achievement (3). The results of the research indicate that physical activity is the basis for the early development of each child and it influences many aspects of the child's health (4). Leading health organizations emphasize that higher level of physical activity in children correlates with the important short-term and long-term health benefits in the physical, emotional, social, and cognitive domains through their whole life. However, it is very important to examine how different programs contribute to the development of different abilities and skills which are necessary for both participation in different sports activities during lifetime and the normal functioning of an individual. One of these programs is yoga for children.

Yoga is a specific form of exercise which is defined as a system of physical positions (asana), breathing techniques (pranayama), and meditations (shavasana), which are practiced with the aim to improve one's physical and emotional well-being. In the pediatrics population, yoga has been used to improve physical fitness, motor skills, strength, negative behavior, attention to task, mindfulness and to reduce stress in older children with typical development and those with physiological disorders and behavioral and developmental conditions (5–7). Areas for which yoga has been studied include physical fitness, cardiorespiratory effects, mental health, behavior and development, irritable bowel syndrome, eating disorders, and prenatal effects on birth outcomes (5). Yoga training became more and more attractive, because it influences the improvement of the health condition in all ages, but the authors state the need for the additional researches on the children population (5).

When it comes to the effects of physical exercises on children's cognitive abilities some research show that yoga practice improves several aspects of cognition and executive functions (8), which can be good predictors of math and reading competence throughout the school years. It is possible that yoga might help improve executive functions, spatial memory scores, strategic planning and the ability to concentrate. It is shown that regular yoga practice is coupled with the improved impulse control, sustained attention, attenuated antisocial and self-harm behaviors, reduced stress, and psychological distress (9), as well as with the positive impact to concentration, attention and behavior (10). These improvements may reflect underlying improvements in motor planning, motor speed, and hand steadiness. In terms of behavior, fine and gross motor skills and the academic achievement, there is an insignificant or no change (8). One of the studies that was conducted exclusively examined the influence of Hatha yoga on the motor abilities, such as strength, flexibility, coordination and balance. When it comes to motor performances, Telles et al. concluded that after the yoga training there is an improvement of statistical performances in children (11), but most of the authors recommended additional research to address methodological shortcomings.

Numerous researches have confirmed that during the physical activity, the level of motor abilities increases, along with development of cognitive abilities. Findings favor causal evidence of relations between physical activity with both motor and cognitive development in preschool children, with increased physical activity having significant beneficial effects on motor abilities and cognitive functioning, but given the small number of studies available in the literature, future research with large representative samples is needed to explore other cognitive domains and to strengthen and confirm the dose-response evidence (1). Regarding that the aim of the study was to examine the effects of yoga on motor and cognitive ablities in preschool children.

## Methods

### Subjects

A total of 45 children (age range 5–6 years) participated in this study for a 12-week period. The sample was divided into two groups: experimental group (*n* = 23) aged 5.82 ± 0.23 years, with a mean height of 115 ± 0.05 cm and a mean weight of 21.45 ± 4.55 kg, and control group (*n* = 22) aged 5.90 ± 0.27 years, with mean height 117 ± 0.05 cm, 22.60 ± 3.60 kg (Table 1).

**Table 1:** Decriptive statistics (Mean ± SD).

**Variable**	**Yoga group**	**Control group**
Number	23	22
Gender	Boys: 9 Girls: 14	Boys: 9 Girls: 13
Age	Initial: 5.82 ± 0.23 Final: 6.06 ± 0.23	Initial: 5.90 ± 0.27 Final: 6.14 ± 0.27
Body weight (kg)	21.43 ± 4.55	22.60 ± 3.60
Body height (m)	1.15 ± 0.05	1.17 ± 0.05
BMI (kg/m^2^)	16.07 ± 2.45	16.42 ± 1.65

All children attended a standard preschool program during the day at one of the preschool institutions in PoŽarevac, Serbia. The experimental group consisted of 23 children who attended the preschool yoga program (three 30-min sessions per week) in addition to their participation in a standard preschool institution program. The control group consisted of 22 healthy children from a single preschool institution who did not engage in any additional organized PA outside of the standard preschool program. It should be noted that the authors point out that in children at this age, weight, height and endurance and development of physical abilities are very similar in girls and boys and that joint participation in the same programs is not contraindicated (12).

All participants' parents gave written consent for their children to participate in the study. This study was approved by the Institutional Review Committee of the University of Niš (Ref. No. 04-1186/2) and was conducted under the Declaration of Helsinki.

### Motor Abilities Assessment

Subtests from the BOT-2test battery were used to evaluate motor abilities (Bruininks-Oseretsky Test of Motor Proficiency). BOT-2 is used as a standardized measure of the level of motor ability in children and adolescents aged 4 to 21 (13). Previous researches in this area have proved that BOT-2 test is valid (14). For the purposes of this research 4 subtests were used: fine motor integration (8 tasks), manual dexterity (5 tasks), balance (9 tasks) and bilateral coordination (7 tasks). The mentioned four subtests include a total of 29 motor tasks, and the last two subtests—balance and bilateral coordination, together give a composite of body coordination, which additionally indicates this ability of the examinee.

### Cognitive Assessment

To evaluate the cognitive abilities of preschool children three subtests of the School Maturity Test (Serbian TZŠ+) were used: visual memory, stacking cubes and codes. The results of the study showed high reliability and validity of this test and they suggest that TZŠ+ has high correlation with the cognitive tests of TYPE1 and with Raven's Colored Matrices (15). *Visual memory* is a test designed to evaluate the ability of memorization and attention span, and it consists of 15 tasks. *Stacking cubes* is a test meant to evaluate the ability of visual-motor coordination, perceptive organization and the ability of planning, and it consists of eight tasks. *Codes* is a test which evaluates the ability of learning from experience, concentration and visual-motor coordination, and it consists of 25 tasks.

For the purposes of this research the school maturity test was ordered and purchased from the “Association of Psychologists of Serbia” from Belgrade. When using this test two psychologists were hired at the Faculty of Sports and Physical Education in Niš and in the “Ljubica Vrebalov” preschool in Požarevac. The hired psychologists conducted the cognitive tests and interpreted the results, which is in accordance with the article 10 of the Rulebook on the Standards and Procedures for the Development and Use of Psychological Measuring Instruments. Testing of all the participants in this experimental study was performed immediatelyy before (the initial measurement) and later again, after the applied experimental exercise programs (the final measurement). All the testings, as well as the implementation of the experimental program was conducted in the gymnasium of “Ljubica Vrebalov” preschool in Požarevac.

### Experimental Program of Yoga Exercises

The duration of the program of exercises is defined on the bases of the recommendations of previous research. In this regard, it was found that the duration of program in most studies ranged between 2 and 4 months, and the treatments ranged most frequently between 1 and 2 h per week, distributed between 2 and 5 h per week. The yoga program was a modified version of standardized yoga curricula for preschool children (16–20). In these curricula most represented kinds of asana are the animal positions (e.g., cat, cow, dog downward) and the positions of the nature (e.g., mountain, tree, moon), because they are relatively simple and interesting to the small children. In accordance with the guidelines that for children under the age of six the total time for yoga session should be up to 20 min, the class was divided into three parts. The introductory part of the class lasted for 5 min and was aimed to prepare the children for the activities in the main part through light breathing exercises. The main part of the class lasted up to 20 min. In this part yoga positions are performed and the children stay in the final position of any yoga asana/exercise for up to 10 sec. This modified form of yoga combines different physical positions, visual images, but it doesn't include extremely deep breathing, because it is considered difficult for small children to include distinct breathing along with holding an appropriate position (8, 21). Final part of the class lasted for up to 5 min and was intended for relaxation.

The 2 × 2 repeated-measures ANOVA was used to compare differences in outcome measures after the intervention period between the two study groups. Moreover, *p*-values of < 0.05 were considered statistically significant. No corrections were applied for multiple comparisons. The data are reported as mean ± SD or frequency (percentage). The data were analyzed using the statistical package SPSS version 20.0 (SPSS Inc., Armonk, NY, USA).

## Results

The results of motor abilities tests indicate certain changes among participants. Improvement in the fine motor integration test results has been noted in favor of the intervention group (*p* = 0.022). A significant differences were detected in the fine motor skills test results (*p* = 0.029), bilateral coordination (*p* = 0.005), balance (*p* = 0.000), and body coordination total score (*p* = 0.000) (Table 2).

**Table 2:** SPANOVA (ANOVA goup × time).

**Variables**	**Yoga**		**Control**		**F**	***p***	**ηp^**2**^**
	**Inicial**	**Final**	**Inicial**	**Final**			
Fine motor integration	12.22 ± 4.56	12.87 ± 3.09	13.41 ± 3.83	11.82 ± 2.26	5.68	0.022^*^	0.117
Manual dexterity	13.70 ± 4.17	16.09 ± 5.51	12.91 ± 4.26	14.00 ± 4.69	0.60	0.445	0.014
Fine motor skills	25.91 ± 7.70	28.96 ± 7.78	26.32 ± 6.16	25.82 ± 5.48	5.10	0.029^*^	0.106
Bilateral Coordination	14.65 ± 2.06	17.22 ± 2.61	16.55 ± 1.99	16.68 ± 2.77	8.69	0.005^*^	0.168
Balance	15.74 ± 4.91	18.17 ± 5.06	15.27 ± 3.01	13.27 ± 3.65	15.60	0.000^*^	0.266
Body Coordination	30.39 ± 5.73	35.39 ± 6.35	31.82 ± 3.32	29.95 ± 5.43	20.44	0.000^*^	0.322
Visual memory	3.22 ± 0.67	3.35 ± 0.49	3.14 ± 0.35	3.27 ± 0.46	0.00	0.975	0.000
Stacking cubes	4.35 ± 0.88	4.48 ± 0.73	4.41 ± 0.67	4.18 ± 0.80	3.02	0.089	0.066
Code	3.43 ± 1.04	4.00 ± 0.85	3.23 ± 0.61	3.41 ± 0.67	2.30	0.137	0.051
Total cognitive Abilities	3.67 ± 0.67	3.94 ± 0.58	3.59 ± 0.40	3.62 ± 0.46	3.56	0.066	0.076

*F-coefficient of the F-test; p—coefficient of significance of the differences*,

* *at the p < 0.05 level; ηp^2^-partial Eta squared*.

## Discussion

This study evaluated the effectiveness of a 12-weeks yoga intervention in preschool children and the results showed that the program had higher influence on motor than on the cognitive abilities. Significant improvements were observed in 5 of total 6 subtests for motor abilities assessment, in fine motor integration, manual dexterity, coordination, and balance, in the intervention group compared to the control group. On the contrary, no progress is observed in 3 subtests for cognitive assessment.

The significant contribution of the experimental program which was recorded on the tests of motor coordination in the variables bilateral coordination, balance and overall body coordination, is in line with previous research (22–25). When it comes to fine motor abilities test, a significant contribution of yoga practice to fine motor integration and overall fine motor abilities has been noted, which is also in line with previous research (8). It is important to point out that motor coordination affects the child's quality of life and different bio-psycho-social aspects (26), and that fine motor abilities have a positive impact on the sensorimotor development of the nervous system (27). Therefore, special attention should be paid to their development in children.

A great improvement noted in the balance subtest is also in line with the results in a large number of previous studies showing that great improvement has been achieved in the static balance of preschool children (28) and school-age children (2, 5, 6, 29). All these findings confirm the theory of motor development in children which says that preschool children, aged 3–6 are in a period of rapid development of balance skills (30). Statistically significant improvement in balance abilities can be attributed to the specific yoga positions. During yoga practice, taking many positions or asanas stimulates the work of the torso stabilizer muscles. This improves the interaction between the upper and lower extremities in the kinetic chain, which facilitates the maintenance of static balance during exercise (31). If this ability isn't developed properly and on time, it will negatively affect other related abilities (30). About 73–87% of children with poor motor development have problems with balance, and these problems have an impact on their further learning of complex motor skills such as climbing, running, cycling etc. (32). These results are consistent with research by Bubel and Gaylord (28), where no progress was made in dynamic balance after a six-month yoga program in preschool children. However, the author emphasizes his assumption that the cause of poor progress in dynamic balance is the weak discriminativeness of BOT-2 items which represent the dynamic balance (items 2 and 5), where the respondents generally achieve high or maximum results, so there is not enough space to quantify progress.

When it comes to cognitive assessment, the contribution of experimental program is not noticed in any of 3 subtests, which is unexpected because the vast majority of previous research suggest slightly different results. A large number of authors claim that the implementation of physical exercise programs for preschool children can significantly contribute to the improvement of their overall psycho-physical development. It can influence the improvement of both their motor abilities and skills and their cognitive abilities, which will enable them to reach their full potential more easily (2, 33–35). We will list the claims of several other significant authors: Lawson et al. (8) suggest that yoga may have positive effects for preschool-age children, particularly in the areas of fine motor and academic performance; Preschool children's fine motor improvement with yoga is consistent, with literature showing fine-motor improvement in school-age children participating in yoga (36, 37); the yoga program helped students feel focused and gave them strategies to control their behavior in stressful situations (38); the significant improvement was observed in measures of mental ability and memory in experimental group of yoga practices in residential school children (39); a positive influence of yoga practice in children on the parameters of visual attention (40); Hillman et al. (41) suggest that the exercise can increase the children's brain volume, improve their cognitive abilities and their academic achievement (41).

Although numerous studies have confirmed that during the physical activity the level of cognitive abilities increases, the result of this study suggest the opposite and indicates that yoga exercise didn't have a significant influence on any variable of cognitive abilities in preschool children. Some studies which agree with the results of this study, although few in number, can still be found, so Songul et al. (42) argue that after 1 h a week of yoga education, significant difference was no found between the pretest and post-test score in averages, which suggests that this topic should be approached with a dose of caution, and that it should be further examined. The tests of coordination are largely related to intelligence, i.e., their adequate performance largely depends on it, and due to this fact it cannot be literally said that there is no progress in cognitive abilities.

Failure to achieve the expected results in our study can be reflected in a small sample, in the insufficient duration of the experimental exercise program itself, which may be the limit of this study, so in future research, it is recommended to use different cognitive tests which will more completely measure a wider range of cognitive abilities. Certainly, we should also keep in mind the analysis of Stojiljković et al. (43), who found that exercise should be added to the simultaneous performance of certain tasks which require cognitive thinking and higher levels of attention. This suggestion should be a guideline for some further research in this field, i.e., for designing a program of yoga exercises that would include solving certain cognitive tasks at the same time.

## Conclusion

This study provides evidence that a 12-week yoga program has beneficial effects on typically developed preschool children, demonstrating a positive influence on their motor abilities. Such research is extremely important, because the preschool period is a critical period for the development of these abilities, and also, the habits that children acquire in this period are reflected in the comprehensive motor development during life.

## Data Availability Statement

The raw data supporting the conclusions of this article will be made available by the authors, without undue reservation.

## Ethics Statement

The studies involving human participants were reviewed and approved by Institutional Review Committee of the University of Niš (Ref. No. 04-1186/2). Written informed consent to participate in this study was provided by the participants' legal guardian/next of kin.

## Author Contributions

AA and BK contributed to conception and design of the study. BK organized the database. BK and BM performed the statistical analysis. AA wrote the first draft of the manuscript. AA, BK, and BM wrote sections of the manuscript. All authors contributed to manuscript revision, read, and approved the submitted version.

## Conflict of Interest

The authors declare that the research was conducted in the absence of any commercial or financial relationships that could be construed as a potential conflict of interest.

## Publisher's Note

All claims expressed in this article are solely those of the authors and do not necessarily represent those of their affiliated organizations, or those of the publisher, the editors and the reviewers. Any product that may be evaluated in this article, or claim that may be made by its manufacturer, is not guaranteed or endorsed by the publisher.

## References

[1] Zeng N Ayyub M Sun H Wen X Xiang P. Effects of physical activity on motor skills and cognitive development in early childhood: a systematic review. Biomed Res Int. (2017) 2760716:1–13. 10.1155/2017/2760716 29387718 PMC5745693

[2] Telles S Singh N Bhardwaj AK Kumar A Balkrishna A. Effect of yoga or physical exercise on physical, cognitive and emotional measures in children: a randomized controlled trial. Child Adolesc Psychiatry Ment Health. (2013) 7:1–16. 10.1186/1753-2000-7-37 24199742 PMC3826528

[3] Strong W Malina RM Blimkie CJ Daniels SR Dishman RK Gutin B. Evidence based physical activity for school-age youth. J Pediatr. (2005) 146:732–7. 10.1016/j.jpeds.2005.01.055 15973308

[4] King G Lawm M King S Rosenbaum P Kertoy MK Young NLA. conceptual model of the factors affecting the recreation and leisure participation of children with disabilities.*Phys Occup Ther Pediatr*.(2003) 23:63–90. 10.1080/J006v23n01_05 12703385

[5] Birdee GS Yeh GY Wayne PM Phillips RS Davis RB Gardiner P. Clinical applications of yoga for the pediatric population: a systematic review. Acad Pediatr. (2009) 9:212–20. 10.1016/j.acap.2009.04.002 19608122 PMC2844096

[6] Donahoe-Fillmore B Grant E. The effects of yoga practice on balance, strength, coordination and flexibility in healthy children aged 10–12 years. J Bodyw Mov Ther. (2019) 23:708–12. 10.1016/j.jbmt.2019.02.007 31733751

[7] Pise V Pradhan B Gharote M. Effect of yoga practices on psycho-motor abilities among intellectually disabled children. J Exerc Rehabil. (2018) 14:581–5. 10.12965/jer.1836290.145 30276177 PMC6165980

[8] Lawson LA Cox J Blackwell AL. Yoga as a classroom intervention for preschoolers. J Occup Ther Sch Early Interv. (2012) 5:126–37. 10.1080/19411243.2012.71375532204774

[9] Kerekes N. Yoga as complementary care for young people placed in juvenile institutions-a study plan. Front Psychiatry. (2021) 12:575147. 10.3389/fpsyt.2021.575147 34149466 PMC8211756

[10] Berger DL Stein RE. Effects of yoga on inner-city children's well-being: a pilot study. Altern Ther Health Med. (2009) 15:36–42. 19771929

[11] Telles S Hanumanthaiah B Nagarathna R Nagendra HR. improvement in static motor performance following yogic training of school children. Percept Mot Skills. (1993) 76:1264–6. 10.2466/pms.1993.76.3c.1264 8337075

[12] Living AH. Prevention of Childhood Obesity Through Increased Physical Activity Council on Sports Medicine and Fitness and Council on School Health. Pediatrics. (2006) 117:1834–42. 10.1542/peds.2006-0472 16651347

[13] Deitz JC Kartin D Kopp K. Review of the Bruininks-Oseretsky test of motor proficiency, (BOT-2). Phys Occup Ther Pediatr. (2007) 27:87–102. 10.1080/J006v27n04_06 18032151

[14] Wilson BN Kaplan BJ Crawford SG Dewey D Dadkhah AE Bruininks RH. Bruininks-oseretsky test of motor proficiency, second edition complete form report other information. Sports Biomech. (2013) 5:2078–273.

[15] Novović Z Tovilović S Jovanović V Biro M. Validacija Testa Zrelosti Za Školu. Primenj Psihol. (2009) 2:129–47. 10.19090/pp.2009.2.129-147

[16] Satyananda SS. Yoga Education for Children: a Manual for Teaching Yoga to Children. Munger, IN: Bihar School Of Yoga (1990).

[17] Komitor J Adamson E. The Complete Idiot's Guide to Yoga with Kids. New York, NY: Penguin Random House (2000).

[18] Wenig M. Yoga Kids: For Ages 3-6. Michigan City: Gaiam-Fitness (2003).

[19] Hester EIL. London: Dorling Kindersley Limited (2006).

[20] Hoffman S. Yoga for Kids. New York, NY: Penguin Random House (2018).

[21] Razza RA Linsner RU Bergen-Cico D Carlson E Reid S. The feasibility and effectiveness of mindful yoga for preschoolers exposed to high levels of trauma. J Child Fam Stud. (2020) 29:82–93. 10.1007/s10826-019-01582-7

[22] Privitellio D Caput-Jogunica S Gulan R Boschi G. The influence of controlled sports activities on motoric capabilities in preschool children. Medicina. (2007) 43:204–9.

[23] Krneta Ž Casals C Bala G Madić D Pavlovic Drid P. Can Kinesiological Activities Change “Pure” motor development in preschool children during one school year? Coll antropol. (2015) 39:35–40. 26434009

[24] Bellows L Davies P Courtney J Gavin W Johnson S Boles R. Motor skill development in low-income, at-risk preschoolers: a community-based longitudinal intervention study. J Sci Med Sport. (2017) 20:997–1002. 10.1016/j.jsams.2017.04.003 28506451

[25] Birnbaum J Geyer C Kirchberg F Manios Y Koletzko B. ToyBox-study Group. Effects of a kindergarten-based, family-involved intervention on motor performance ability in 3-to 6-year-old children: the ToyBox-study. J Sports Sci. (2017) 35:377–84. 10.1080/02640414.2016.1166390 27033346

[26] Silva P Gabbard SC Ries C Bobbio LGK Interlimb TG. Coordination and academic performance in elementary school children. Pediatr Int. (2016) 58:967–73. 10.1111/ped.12972 26940287

[27] Ivković M Milanović S Velinov N Nikolić D. “Fonetska gimnastika,” in Požarevac: Ustanova za predškolsko obrazovanje i vaspitanje dece Ljubica Vrebalov (2004).

[28] Bubela D Gaylord S. A comparison of preschoolers' motor abilities before and after a 6 week yoga program. J Yoga Phys Ther. (2014) 4:1–4. 10.4172/2157-7595.100015

[29] Mykhno LS Loza TO. Effectiveness of yoga-aerobic means' application in physical education of primary school pupils. Pedagog psihol med-biol probl fiz vihov sportu. (2016) 20:35–40. 10.15561/18189172.2016.0105

[30] Jiang GP Jiao XB Wu SK Liu JiZQ Chen WT Wang X. proprioception, and gross motor development of chinese children aged 3 to 6 years. J Mot Behav. (2018) 50:343–52. 10.1080/00222895.2017.1363694 28915098

[31] Ni M Mooney K Balachandran K Richards A Harriell L Signorile K. utilization patterns vary by skill levels of the practitioners across specific yoga poses (asanas). Complement Ther Med. (2014) 22:662–69. 10.1016/j.ctim.2014.06.006 25146071

[32] Monbarg R Jelsma D Hartman E. Effect of Wii-intervention on balance of children with poor motor perfor-mance. Res Dev Disabil. (2013) 34:2996–3003. 10.1016/j.ridd.2013.06.008 23827983

[33] Graham G Parker M. Children Moving. New York, NY: McGraw-Hill Publishing (2013).

[34] Raghuraj P Nagarathna R Nagendra HR Telles S. Pranayama increases grip strength without lateralized effects. Indian J Physiol Pharmacol. (1997) 41:129–33. 9142556

[35] Slovacek SP Tucker SA Pantoja LA. Study of the Yoga ed Program at the Accelerated School. Program, Evaluation, and Research, Collaborative Charter College of Education: Los Angeles (2003).

[36] Dash M Telles S. Yoga training and motor speed based on a finger tapping task. Indian J Physiol Pharmacol. (1999) 43:458–62. 10776461

[37] Manjunath NK Telles S. Spatial and verbal memory test scores following yoga and fine arts camps for school children. Indian J Physiol Pharmacol. (2004) 48:353–6. 15648409

[38] Case-Smith J Sines JS Klatt M. Perceptions of children who participated in a school-based yoga program. J Occup Ther Sch Early Interv. (2010) 3:226–238. 10.1080/19411243.2010.520246

[39] Verma A Shete SU. Singh thaku G. The effect of yoga practices on cognitive development in rural residential school children in India. Memory. (2014) 6:6–24. 10.NJLM/2014/8281:2015

[40] Jarraya S Wagner M Jarraya M Engel FA. 12 weeks of Kindergarten-based yoga practice increases visual attention, visual-motor precision and decreases behavior of inattention and hyperactivity in 5-year-old children. Front Psychol. (2019) 10:796–811. 10.3389/fpsyg.2019.00796 31024412 PMC6467975

[41] Hillman CH Pontifex MB Raine LB Castelli DM Hall EE. The effect of acute treadmill walking on cognitive control and academic achievement in preadolescent children. Neuroscience. (2009) 159:1044–54. 10.1016/j.neuroscience.2009.01.057 19356688 PMC2667807

[42] Songül YÖ Özkul B Oral E Semin I. The Effects Of Yoga Education On The Cognitive Functions Of Children In Early Childhood. Egitim ve Bilim. (2021) 46:303. 10.15390/EB.2020.908832932771

[43] Stojiljković N Mitić P Sporiš G. Can exercise make our children smarter? Ann Kinesiol. (2020) 10:115–27. 10.35469/ak.2019.211

